# Commentary: A Gene-Based Association Method for Mapping Traits Using Reference Transcriptome Data

**DOI:** 10.3389/fpsyt.2016.00073

**Published:** 2016-04-25

**Authors:** Udi E. Ghitza

**Affiliations:** ^1^U.S. Department of Health and Human Services, Center for the Clinical Trials Network, National Institute on Drug Abuse, National Institutes of Health, Bethesda, MD, USA

**Keywords:** genetics, gene expression, bioinformatics, informatics, precision medicine, addiction, drug abuse, substance-use disorders

Uncovering and understanding links between gene expression, brain structure and function, and behavior may inform precision medicine by identifying more precise strategies to prevent and treat disease. Indeed, gene expression regulation mechanisms contribute to disease susceptibility, drug response, and the course of many substance-use disorders (1–3). Gamazon et al. recently reported development of an innovative gene-based association method to further understanding of gene-expression regulation mechanisms mediating relationships between single nucleotide polymorphism (SNP) variations and phenotypic variability of complex traits (4). They developed a computational method to utilize publically available gene expression datasets to estimate proportion of gene expression influenced by a person’s genetic profile – genetically regulated expression – and to correlate this with complex phenotypes under investigation. Their whole-genome tissue-dependent prediction models – incorporating information on gene expression regulation from a set of markers in large high-resolution transcriptome databases – may be used to uncover mechanisms by which SNP-regulated gene expression contributes to disease susceptibility and complex traits. The investigators harnessed the power of large reference transcriptome data sets, such as the Genotype-Tissue Expression (GTEx) Project, the Genetic European Variation in Health and Disease (GEUVADIS), and Depression Genes and Networks (DGN), among others – in which both gene variation and gene expression levels have been measured – to estimate genetically regulated gene expression of SNPs. This information and genome-wide association study (GWAS) data are used to compose additive models of gene expression traits trained in reference transcriptome data sets. Thus, this novel method – termed PrediXcan – utilizes predictive algorithms to correlate associations between estimated genetically regulated gene expression of SNPs and phenotypic traits of interest using regression methods and non-parametric approaches, as a way to powerfully identify disease risk-mediating and trait-associated genes. Interestingly, unlike other gene-based testing methods, PrediXcan allows researchers to ascertain the direction of these associations. The authors demonstrated the utility of their method by identifying and replicating numerous new candidate associations within a previous data set. They explain how such an approach may be used to increase statistical power in genetics studies by reducing the multiple-testing penalty, which burdens many single-variant analysis approaches. Therefore, this method may afford researchers greater capability of reusing GWAS or whole-genome sequence data sets to detect novel trait-associated loci explaining a large portion of disease susceptibility-associated phenotypic variability under control of genetically regulated gene expression. Research is needed to improve prediction capabilities of this approach when applying it to substance-use disorders and other behavioral health disorders, and to broaden its utility to map links between epigenetics and disease risks or traits.

There are many evidence gaps regarding how gene-regulation mechanisms may interact with environmental risk factors to contribute to disease susceptibility for substance-use disorders, especially during periods of high vulnerability such as brain and cognitive developmental windows during youth and adolescence (5). Therefore, a high-priority area for future research may be to employ this PrediXcan method or similar computational gene-testing approaches to identify human gene-expression-regulated genetic factors and mechanisms mediating risk and resilience for drug misuse and substance-use disorders, for instance, during brain and cognitive development. As the authors suggest, reanalyzing existing publically available GWAS data sets in comprehensive biorepositories may increase the efficiency and cost-efficiency of elucidating such mechanisms (4). Another high-priority precision-medicine area is to determine whether and how prediction of expression profiles derived from genetic variance may be applied to different patient subgroups (e.g., those without substance-use problems, versus those exhibiting unhealthy drug or alcohol use but not yet considered to be on the severe end of the spectrum, versus those patients with severe substance-use disorder).

For this research to include phenotypes informative to both addiction-medicine researchers and clinical practice providers, rigorous and systematic research first needs to identify patient-centered health outcomes, which are reliably correlated with clinically meaningful changes in drug use in different populations and settings of patients with substance-use disorders. This would be particularly helpful given that substance-use disorders are chronic conditions often characterized by cycles of use, reduced use, abstinence, and relapse to use (6–8). Rigorous systematic research is also needed to establish reliable biomarkers for substance abuse, which might be used as objective measures for guiding such genetics research to predict risk for developing substance-use disorders, response to treatment, and risk for relapse. Thus, science needs to establish widely accepted clinically relevant phenotypic endpoints and biomarkers, which may help in standardizing data collection and enable development of common data elements (CDEs) of clinically relevant phenotypic measures across studies. CDEs may, in turn, facilitate exchange of data by precisely describing semantic characteristics for a discrete piece of data, which will be collected, stored, or exchanged during the course of a study. CDEs of such measures could be key tools in building scalability in which uniform data elements with common semantic characteristics collected across studies can be incorporated and exchanged across networks and systems of networks. These benefits may be enhanced when CDEs conform to well-accepted data standards and ontologies (9). Thus, development and use of CDEs of clinically relevant phenotypic measures may facilitate this line of research by improving the efficiency and quality of data collection, as well as cross study comparisons, data aggregation, and meta-analyses.

Collectively, the above research initiatives are needed to accelerate the capacity to catalog gene expression mechanisms by which genetic variations map onto addiction risks and altered brain maturation, brain circuit function, and substance-use disorder behavioral patterns. Uncovering strong genetic evidence for such mechanisms can then be leveraged to develop an evidence-based Research Domain Criteria (RDoC) framework for substance-use disorder phenotypes, to complement one already established for other psychiatric conditions by the U.S. National Institute of Mental Health (NIMH) (10). Developing such RDoCs would expedite mechanistic precision-medicine research on genetics of substance-use disorders and psychiatric comorbidity to inform development of evidence-based approaches to improving patient-centered care for patients with multiple co-occurring psychiatric conditions.

## Author Contributions

UG, Ph.D., undertook a review of the literature, conceived of this general commentary, and wrote and reviewed all drafts.

## Disclaimer

The opinions in this paper are those of UG and do not represent the official position of the U.S. government.

## Conflict of Interest Statement

The author declares that the research was conducted in the absence of any commercial or financial relationships that could be construed as a potential conflict of interest.
